# Parliament and the Profession

**Published:** 1918-02-09

**Authors:** 


### Parliament and the Profession.

### Venereal Poisons.

Mr. Macpherson informed Mr. Peto that there is no Army Order authorising or forbidding the use of prophylactic medical measures.

### Hospitals in School Buildings.

Mr. Shirley Benn asked the Under Secretary of State for War what steps are being taken by the military to vacate school buildings and adopt other buildings for hospitals, and if he was aware that in Plymouth, owing to the commandeering of certain schools by the Army and the Admiralty, there are 9,041 children working on the double shift plan, and only doing in three years what was formerly done in two. Mr. Macpherson said that the desirability of surrendering these schools is recognised, and a promise has been given that they shall be surrendered as soon as the military authorities can arrange to forego their use.

### Director of Medical Service (Ministry of Pensions).

In reply to a question asked by Sir G. Thomas, Sir. A. Griffith-Boscawen said that the Director of Medical Services to the Ministry of Pensions is entrusted with the administrative control of the Chelsea Medical Board, of the Special Medical Boards in London and the provinces, and of the other medical services of the Ministry, and also acts as adviser in matters of treatment and in the establishment and supervision of institutions for this purpose. The appointment (which is for no specified period) is a whole-time one, carrying a non-pensionable salary of £1,200 a year, the amount to be subject to reconsideration after the war. In the case, however, of the present holder, Sir John Collie, the Treasury have, at the request of the Ministry, sanctioned the payment of a salary personal to himself of £1,500 a year.

